# Change in Growth and Diet Quality Among Preschool Children in Tokyo, Japan

**DOI:** 10.3390/nu12051290

**Published:** 2020-05-01

**Authors:** Chisa Shinsugi, Yukako Tani, Kayo Kurotani, Hidemi Takimoto, Manami Ochi, Takeo Fujiwara

**Affiliations:** 1 National Institute of Health and Nutrition, National Institutes of Biomedical Innovation, Health and Nutrition, 1-23-1 Toyama, Shinjuku-ku, Tokyo 162-8636, Japan; shinsugi@nibiohn.go.jp (C.S.); kurotani@nibiohn.go.jp (K.K.); thidemi@nibiohn.go.jp (H.T.); 2 Department of Global Health Promotion, Tokyo Medical and Dental University, 1-5-45 Yushima, Bunkyo-ku, Tokyo 113-8519, Japan; tani.hlth@tmd.ac.jp; 3 Department of Health and Welfare Services, National Institute of Public Health, 2-3-6, Wako, Saitama 351-0197, Japan; ochi.m.aa@niph.go.jp

**Keywords:** child growth, diet quality, Japanese Food Guide Spinning Top score (JFGST score), brief-type self-administered diet history questionnaire (BDHQ), preschool children, Japan

## Abstract

Dietary intake of adequate quality and quantity in early life is essential for healthy growth and development. This study aimed to examine the association between one-year change in growth and diet quality in preschool children in Adachi City, Tokyo, Japan. A total of 110 participants (49% boys, four to five years old at baseline) were included in this analysis. Body mass index for age z-score (BAZ) and height for age z-score (HAZ) were calculated in accordance with WHO reference. Dietary intakes were assessed using the brief-type self-administered diet history questionnaire for children (BDHQ3y), and daily quality score was calculated based on the Japanese Food Guide Spinning Top (JFGST score). Regression analyses found no significant association between one-year change in growth and diet quality (compared to a low JFGST score, BAZ: β = 0.16, 95% CI: −0.29 to 0.60 for a middle JFGST score, β = −0.14, 95% CI: −0.61 to 0.33 for a high JFGST score, HAZ: β = −0.15, 95% CI: −0.50 to 0.21 for a middle JFGST score, β = −0.06, 95% CI: −0.43 to 0.30 for a high JFGST score). Further studies are needed to develop an appropriate diet quality index for healthy growth in Japanese preschool children.

## 1. Introduction

Child malnutrition is a serious public health concern globally [1,2]. The World Health Organization stated that malnutrition refers to deficiencies, excesses, or imbalances in a person’s intake of energy and/or nutrients, and is split into three broad groups of conditions: undernutrition (indicating stunting and underweight), micronutrient-related malnutrition (including micronutrient deficiencies), and overnutrition (indicating overweight and obesity) [3]. Undernutrition, including stunting and vitamin A and zinc deficiency, was estimated to cause 45% of all child deaths in 2011 globally [4], and the prevalence of stunting was estimated at 6% in developed countries [2]. In a population-based study of fourth grade school children in Japan, the prevalence of underweight was approximately 10% [5] and increasing trends of underweight among adolescent girls were observed [6]. Undernutrition in early life leads to the risk of chronic diseases including high glucose concentrations, weaker academic performance, and poorer economic productivity, such as income and household assets [7]. The prevalence of childhood overweight and obesity is increasing worldwide and is estimated at 25% in OECD countries [8]. In Japan, the prevalence of overweight among preschool children was estimated at 13.4% [9] and there were increasing trends of obesity among children aged 6–14 years old [10].

Adverse health impacts of childhood obesity have been revealed across the lifespan [11,12,13]. Early childhood is a critical period for healthy growth and development of the child’s body, and regular assessment of linear growth of adequate weight gain and height progression for age are essential [14]. Diet may be an important target to prevent this impaired child growth, and intervention effects of feeding on promoting healthy growth in early childhood are suggested [14,15]; however, it remains unclear what quality and quantity of dietary intake is necessary for healthy growth and development in early life. 

Several studies have suggested that certain foods and nutrient intake may influence child growth [16,17,18,19,20,21]; micronutrient deficiency including iron and zinc may affect growth retardation or stunting [16]. Moreover, a systematic review indicated that higher protein intake in infancy and early childhood is associated with increased growth [20] but may contribute to increased risk for later obesity. Another systematic review revealed that overweight children with greater fruit and vegetable consumption were less likely to remain overweight, but this review study concluded the association remains unclear [21]. Therefore, the appropriate amount of each food may be important since a high intake of particular foods can lead to unbalanced diets and eventually result in overweight or obesity. Along with the specific foods and nutrient intake, habitual eating behavior on body weight has been discussed [22]. In particular, some form of problematic eating behavior such as picky eaters in young children may be linked to imbalanced habitual dietary intake. Picky eaters among Canadian preschool children consumed less energy and less protein and were more likely to consume less than the dietary recommendations for fruit, vegetables, and meat [23]. Because children do not grow up eating only specific foods, and because nutrients interact with each other and affect bioavailability [24], capturing the whole dietary intake from habitual eating behavior is useful when considering growth management. Hence, instead of the dominant approach of focusing on single nutrients or foods, the approach of evaluating the overall diet comprehensively is increasingly being adopted [24]. 

Diet quality index has increasingly been used to assess overall dietary intake, and the impact of diet quality on child growth have been investigated in several studies [25,26,27]. A systematic review indicated that low adherence to a Mediterranean-like diet was related with being overweight and obese in European preschool children [25]. In Australian overweight school children, the improvement of diet quality, assessed by adherence to the 2003 Australian Dietary Guidelines for Children and Adolescents, was a lower gain in body mass index (BMI) z-scores (zBMI) [26]. In Dutch girls, on the other hand, better diet quality, reflecting adherence to Dutch Guidelines for a Healthy Diet of 2015, was associated with increased height and weight, which was explained by a higher fat-free mass, not fat mass or percentage body fat, at the age of eight years [27]. However, little is known about the impact of diet quality on child growth in Asian countries. 

In Japan, a diet quality index for adults was developed as a scoring of adherence to the Japanese Dietary Guidelines, and the association of higher diet quality with lower risk of mortality and smaller waist circumference was observed in adults and young women [28,29]. The recommended quantity of dietary intake for those six years of age and older was established as the Japanese Food Guide Spinning Top (JFGST) by the government in July 2005 to guide what kinds of and how much food they should eat in a day to promote health [30]. For children below six years, some dietary experts have developed the recommended quantity, taking account into the estimated energy requirement and the expectation of eating more vegetables in the practical setting [31]. However, to our knowledge, no study has examined the association between diet quality using the JFGST and growth among Japanese preschool children using a longitudinal survey. The purpose of this study was to examine the relationship between diet quality and one-year changes in growth among preschool children in Adachi City, Tokyo, Japan.

## 2. Materials and Methods

### 2.1. Study Design and Participants

We used data on the dietary habits for preschool children performed in 2016 for the baseline survey (T1) and in 2017 for the follow-up survey (T2). Among the public preschools in Adachi City, Tokyo, Japan, a convenience sample of seven public preschools agreed to cooperate with this study. All children in classes with 4-year-old children in these preschools were invited to participate in the baseline survey.

**Figure 1:**
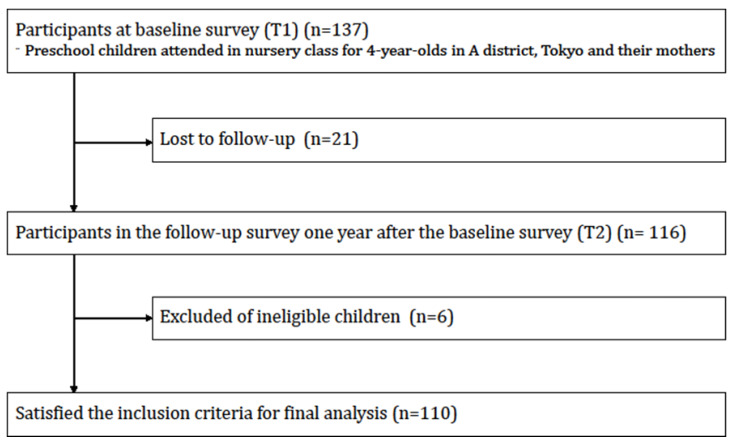
Participants flow chart for analytic sample.

A questionnaire on dietary and lifestyle characteristics was distributed to 154 guardians of preschool children and 137 (response rate: 89%) completed the questionnaire during January and February 2016. Of those, 116 participants completed a one-year follow-up survey (follow-up rate: 84%) and a total of 110 preschool children (54 boys and 56 girls, 4–5 years old at baseline) were included in the analysis after excluding ineligible observations: one child who had outlying data on the outcome variables (BMI for age z-score (BAZ) < −5), and five children who had incorrect values of age and sex (age did not match actual study period, gender mismatch at baseline and follow-up). A participant flow chart is shown in Figure 1. The study was carried out by Adachi City and the use of the secondary data was approved by the Institutional Review Board of the Tokyo Medical and Dental University (approval number M2016-284-02). Prior to data collection, the nursery school principals explained the scope and details of the survey to the participants. Those who opted not to participate in the surveys could decline to respond. Consent to the surveys was confirmed by submitting the questionnaire that the participants answered. 

### 2.2. Anthropometric Data 

Children’s height and weight were reported by their guardians at both T1 and T2 via a questionnaire. BAZ and height-for-age z-score (HAZ) were calculated in accordance with the WHO 2006 and 2007 growth reference standards [32,33]. Growth status of change in BAZ (T2-T1) and change in HAZ (T2-T1) were defined as dependent variables.

### 2.3. Dietary Intake Data

Dietary intake data were collected using a diet history questionnaire for Japanese preschool children aged 3 to 6 years (BDHQ3y) reported by their guardians at T1. Guardians, not employees of preschools reported the dietary intake of their children for both at home and in the preschool. On weekdays, children ate breakfast and dinner at home, and lunch and snacks at the preschools. All of the public preschools which participated in this study provided lunch, as well as snacks between meals. The daily food menus of the meals and snacks were distributed to each family; therefore, guardians knew the dietary intake of their children in the preschool. The BDHQ3y contains 69 food items, which are commonly consumed in Japan, and investigates the consumption frequency of the selected foods and dietary behavior over the preceding month; its validity was assessed [34]. Daily intake of energy and 42 selected nutrients were estimated based on the Standard Tables of Food Composition in Japan [35]. 

Diet quality score of adherence to the JFGST was developed based on a study by Kurotani et al. [28] and generated using information in the daily dietary intake (Table 1). In brief, the JFGST provides serving standards in five dish categories: grain dishes, vegetable dishes, fish and meat dishes (meat, fish, egg and soy-bean dishes), milk (milk and milk products), and fruits. Supplementary Table S1 shows the foods in BDHQ3y that are classified into the five dish categories [36]. One serving for each dish category is defined as follows: One serving of grain dishes contains about 40 g carbohydrates from foods classified as grain dishes. The main ingredient in one serving of vegetable dishes weighs about 70 g of foods classified as vegetable dishes. One serving of fish and meat dishes contains about 6 g protein from foods classified as fish and meat dishes. One serving of milk contains about 100 mg calcium from foods classified as milk dish category. The main ingredient in one serving of fruits weighs about 100 g of foods classified as fruits dish category. The amount of energy intake from snacks and beverages is recommended as less than 200 kcal/day. Because the JFGST established by the Japanese government does not have the recommended amount of servings for each dish category for children under 6 years old, we used the one created by Tokyo Metropolitan Government as an alternative [31]. The recommended total energy intake was based on “Dietary Reference Intakes for Japanese (2015)” [37]. 

The JFGST score ranged from 0 (the lowest diet quality) to 70 (the highest) and was obtained from summing the scores of seven items, namely grain dishes, vegetable dishes, fish and meat dishes, milk, fruits, total energy intake, and energy from snacks and beverages. If a child consumed the recommended amount of servings from any of the five dish categories or the recommended total energy, or energy from snacks and beverages, 10 points were recorded for him/her. If a child exceeded or fell short of the recommended servings or energy, the score was calculated proportionately between 0 and 10 points. If a child consumed less than the recommended amount of servings or energy, the score was calculated using the following formula: 10 × (the consumed amount of servings or energy)/(the lower limit of the recommended amount). If a child consumed more than the recommended amount of servings or energy, the score was calculated using the following formula: 10 − 10 × [(the consumed amount of servings or energy) − (the upper limit of the recommended amount)]/(the upper limit of the recommended amount). For vegetable dishes and fruits, we removed the upper limit of intake. Each score was rounded off to the nearest whole number. When this calculation produced a negative score due to excess servings or energy, the score was converted to 0. The JFGST score and dietary intake of each dish and item were divided into tertiles (low, middle, and high). The JFGST score was defined as an independent variable.

### 2.4. Statistical Analysis

Chi-square test for categorical variables and trend test for continuous variables were performed to examine the differences in demographic data according to the JFGST score at baseline (T1). A regression model was used to estimate the association between JFGST score and growth status. In addition, a regression model was used to estimate the association between dietary intake of each dish (T1) and change in BAZ and HAZ (T2-T1) after adjustments of all other dish categories as covariates. *P* values less than 0.05 were considered statistically significant in all analyses. Statistical analyses were performed with the Stata statistical software Macro Package version 15.1 (Stata Corporation, TX, USA). 

## 3. Results

The demographic characteristics of the participants are shown in Table 2. The mean age of the children was 4.8 years (SD: 0.4). The mean BAZ and HAZ at baseline were 0.21 and −0.77, respectively. The percentages of mild thinness and stunting were 4.6% and 10.0%, respectively, while the percentage of overweight including obesity was 14.6%. 

Table 3 presents the distribution of the scores on the adherence to the JFGST and the intakes of each dish category according to the JFGST score. The mean adherence to the JFGST score was 55.7.

Table 4 shows the result of regression analyses for association between JFGST score at baseline (T1) and change in BAZ and HAZ (T2-T1). No statistically significant differences in change in BAZ and HAZ were observed according to the JFGST score (low, middle, and high). Compared to a low JFGST score, BAZ: β = 0.16, 95% CI: −0.29 to 0.60 for a middle JFGST score, β = −0.14, 95% CI: −0.61 to 0.33 for a high JFGST score, HAZ: β = −0.15, 95% CI: −0.50 to 0.21 for a middle JFGST score, β = −0.06, 95% CI: −0.43 to 0.30 for a high JFGST score.

Table 5 describes the association between dietary intake of each dish at baseline (T1) and change in BAZ and HAZ (T2-T1) adjusted for dietary intake of all dish. High intake of total energy (β = 0.56, 95% CI: 0.02 to 1.10) was positively associated with change in BAZ compared to a low intake. High intake of fish and meat dishes (β = −0.53, 95% CI: −0.97 to −0.09) was inversely related to change in HAZ compared to a low intake.

## 4. Discussion

This study investigated the association between diet quality and change in growth status among preschool children in Japan. Impaired child growth was observed: the proportion who were overweight including obesity was 14.6%, while the proportion who were stunted was 10.0% according to WHO criteria. No significant relationship between one-year change in growth status and diet quality score defined by adherence to the JFGST was observed in this study. Intake of total energy was positively associated with child growth calculated by BAZ after adjustment for other dishes.

In contrast to previous studies [26,27], diet quality was not associated with child growth. Notably, the components of the dietary guideline index in the previous studies were different from those used in this study; for example, nuts or oils and fats were included in the Dutch score, and frequency of water consumption is one component in the Australian index. In the Dutch score, healthier choices were taken into account within the components (for example, vegetable oils rather than total fat). In our study, however, the category of fish and meat dishes in JFGST does not identify the difference in fat quality, that is, fish (rich in polyunsaturated fatty acid) and meat (rich in saturated fatty acid) are treated equally in fish and meat dishes. In preschoolers who participated in the Special Supplementation Nutrition Program for Women, Infants, and Children, intake of water instead of sugar–sweetened beverages was encouraged in order to decrease the risk of overweight [38]. Thus, the formulation of a nutritious diet for preschoolers should be discussed further.

Another study used a diet quality score characterized by frequent consumption of fruit, vegetables, and fish identified by a principal component analysis. The findings from this study suggested that a lower diet quality score was strongly associated with a higher fat mass z-score, but not BMI z-score in children in the UK aged six years [39]. In other words, adiposity accumulation, instead of overall nutritional status, may be more affected by diet quality in early childhood. 

Another possible explanation for the lack of association between diet quality and child growth may be the distribution of JFGST scores. Low intake of vegetable dishes and high intake of snacks and beverages may contribute to the low JFGST score (Table 3). This means that there was no significant difference in intake of dish categories from grains and animal-source foods (fish and meat, milk), which are related to better child growth [40]. Moreover, since the children categorized in the middle and high groups of JFGST score at baseline had less thinness and obesity, it may be difficult to capture the changes in child growth after one year. 

Higher intakes of total energy overall across all children in the classes of four-year-old children led to increased BAZ after one year. However, we understand the limitation in obtaining absolute values. As with other food frequency questionnaires, it is difficult to estimate energy intake with BDHQ3y [34]. Nonetheless, a positive association between energy intake and BMI z-score has been reported in two- to nine-year-old European children [41]. Hence, further research using more accurate nutrition assessment tools is required.

One possibility for the negative association between consumption of fish and meat dishes and HAZ may be time-lag bias, that is, the impact of consuming fish and meat dishes among children in the classes of four-year-old children might appear much later. Moreover, meat and fish intake has a low correlation with estimated meat and fish intake from dietary records and may not be properly measured [34]. Further studies between dietary intakes and growth in height in later childhood including in the development of secondary sexual characteristics are therefore needed.

Several limitations of this study need to be noted. First, the participants were selected from just one city of Tokyo, and hence it is not a nationally representative sample. Thus, our results cannot be generalized since selection bias may be present. Second, because guardians reported dietary intake of their children, there may be errors in the estimation of dietary intake. This may occur when the dietary intake of the children was in the preschool even though the daily food menu of school meals and snacks were provided. Another possible source of error is in the estimation of the height and weight of the participants. This was reported by the guardians. We could not take into account differences in measuring equipment. A systematic review showed that parents were likely to underestimate the weight of their overweight children [42]. However, regular health checkups, including height and weight measurements, are performed in the preschool and the results are reported to the participants and their guardians. Third, the BDHQ3y carries low validity for energy intake and the intake of some foods (e.g., potato and meat intake) [34]. Therefore, the relationship between diet quality and child growth with regard to energy and meat and fish intakes may not have been accurately assessed. Although any self-reported dietary assessments could be susceptible to measurement errors [43], habitual intakes of the participants could be captured in this study. Fourth, we calculated the diet quality score using the JFGST. This clear and practical tool was established in order to enable Japanese people to understand what constitutes a healthy diet in daily life [30]. The calculation methods for the diet quality score for adults have been examined in several studies [28,29]. Due to a lack of national consensus on the threshold of each dish and energy for children under six years old, we instead utilized the criteria created by Tokyo Metropolitan Government [30]. To our knowledge, this is the first study to explore the diet quality of Japanese preschoolers. Nevertheless, further studies are necessary for the validation of the dietary reference quantities in early childhood. Fifth, we cannot completely eliminate the effects of potential confounding factors including socioeconomic status, parental nutritional knowledge and educational status. Sixth, the small sample size of this study must be noted. Finally, although this study was of a longitudinal design, potential confounding factors including residual and unmeasured variables could not be ruled out completely.

## 5. Conclusions

This longitudinal study of Japanese preschool children showed no association between overall diet quality score using the JFGST and growth measured by change in BAZ and HAZ. These findings imply that the development of appropriate quality and quantity references in early childhood is necessary for healthy growth, and the promotion and implementation of a well-balanced diet would be useful in any food environment including at home and in nursery facilities.

## Figures and Tables

**Table 1:** Components of the Japanese Food Guide Spinning Top score in preschool children (0–70).

Component	Recommended Amount of Servings/Energy	Standard for Minimum Score of 0 Points	Standard for Continuous Scoring of 0–10 Points	Standard for Maximum Score of 10 Points
Grain dishes (serving/d) ^a^	3–4	0	>0–<2.5 or ≥4.5	2.5–<4.5
Vegetable dishes (serving/d) ^b^	4	0	>0–<3.5	≥3.5
Fish and meat dishes (serving/d) ^c^	3	0	>0–<2.5 or ≥3.5	2.5–<3.5
Milk (serving/d) ^d^	2	0	>0–<1.5 or ≥2.5	1.5–<2.5
Fruits (serving/d) ^e^	1–2	0	>0–<0.5	≥0.5
Total energy (kcal/d)				
Boy	1300		<1250 or >1350	1250–1350
Girl	1250		<1200 or >1300	1200–1300
Snacks and beverages (kcal/d)	<200	-	≥200	0–<200

A total Japanese Food Guide Spinning Top score (0–70) = grain score + vegetable dishes score + fish and meat dishes score + milk score + fruits score + total energy score + snacks score. ^a^ One serving contains about 40 g carbohydrates from foods classified as grain dishes. ^b^ In one serving, the main ingredient weighs about 70 g from foods classified as vegetable dishes. ^c^ One serving contains about 6 g protein from foods classified as fish and meat dishes. ^d^ One serving contains about 100 mg calcium from foods classified as milk dish category. ^e^ One serving of the main ingredient weighs about 100 g from foods classified as fruits dish category.

**Table 2:** Characteristics of the study population at baseline (T1) (*n* = 110).

Variable	JFGST Score at Baseline (T1)								
	Total (n = 110)		Low (34.55%)		Middle (35.45%)		High (30.0%)		*p*
	Mean/%	SD	Mean/%	SD	Mean/%	SD	Mean/%	SD	
Age (years)	4.8	0.4	4.9	0.3	4.8	0.4	4.7	0.5	0.04
Sex (Boy, %)	49.1		52.6		48.7		45.5		0.83
Height (cm)	107.9	6.0	108.7	6.5	108.6	6.0	106.4	5.2	0.08
Weight (kg)	18.3	2.4	18.5	2.8	18.4	2.3	17.8	1.8	0.61
BAZ	0.2	0.9	0.2	1.0	0.2	0.7	0.3	1.0	0.54
BAZ (%)									
Severe and mild thinness (<−1SD, %)	4.6		10.5		0.0		3.0		0.13
Normal (−1SD≤ to ≤+1SD, %)	80.9		76.3		89.7		75.8		
Overweight and obesity (>+1SD, %)	14.6		13.2		10.3		21.2		
Obesity (>+2SD, %)	3.6		7.9		0.0		3.0		
HAZ	−0.8	1.2	−0.7	1.2	−0.7	1.1	−0.9	1.2	0.28
HAZ (%)									
Stunting (<−2SD, %)	10.0		10.5		7.7		12.1		0.82
Whether child has food allergies (%)									
Yes	12.7		15.8		12.8		9.1		0.80
No	85.5		81.6		84.6		90.9		
Other/missing	1.8		2.6		2.6		0		
Playing (%)									
Very active	61.8		65.8		59.0		60.6		0.53
Active	16.4		10.5		23.1		15.2		
Normal	17.3		15.8		12.8		24.2		
Inactive	3.6		5.3		5.1		0		
Missing	0.9		2.6		0		0		
Caregiver’s employment (%)									
Self-employed	6.4		13.2		2.6		3.0		0.10
Full-time job	49.1		39.5		46.2		63.6		
Part-time job	37.3		44.7		43.6		21.2		
Others / missing	7.3		2.6		7.7		12.1		

BAZ, body mass index for age z-score; HAZ, height for age z-score; JFGST score, Japanese Food Guide Spinning Top score; Chi-square test for categorical data and trend test for continuous variables.

**Table 3:** Distribution of the scores on the adherence to the Japanese Food Guide Spinning Top (JFGST) and the intakes of each dish category according to the JFGST score at baseline (T1) (*n* = 110).

Variable	JFGST Score at Baseline (T1)								
	Total (n = 110)		Low (34.55%)		Middle (35.45%)		High (30.0%)		*p*
	Mean	SD	Mean	SD	Mean	SD	Mean	SD	
JFGST score (0–70)	55.7	7.5	47.6	5.1	56.5	2.0	64.1	2.6	<0.001
Each component score:									
Grain dishes (0–10)	9.3	1.5	8.7	2.2	9.5	0.9	9.7	0.8	0.05
Vegetable dishes (0–10)	6.2	2.5	4.9	2.2	6.2	2.3	7.8	2.4	<0.001
Fish and meat dishes (0–10)	8.4	2.2	7.8	2.6	8.6	2.2	8.9	1.5	0.10
Milk (0–10)	6.8	3.4	5.2	3.3	6.6	3.6	8.9	1.8	<0.001
Fruits (0–10)	8.7	2.2	8.1	2.3	8.3	2.6	9.8	0.6	<0.001
Total energy (0–10)	8.0	2.1	6.7	2.5	8.3	1.8	9.1	1.0	<0.001
Snacks and beverages (0–10)	8.3	3.2	6.2	4.1	9.1	2.2	10.0	0.2	<0.001
Intakes of dish categories (/day)									
Grain dishes (serving)	2.8	0.8	2.7	1.0	2.8	0.7	2.9	0.6	0.55
Vegetable dishes (serving)	2.4	1.5	1.7	0.8	2.3	1.4	3.3	1.8	<0.001
Fish and meat dishes (serving)	3.4	1.2	3.2	1.4	3.4	1.2	3.5	0.8	0.05
Milk (serving)	2.1	1.5	2.3	2.1	2.1	1.5	2.0	0.7	0.61
Fruits (serving)	0.7	0.4	0.6	0.4	0.6	0.3	0.8	0.4	<0.01
Total energy intake (kcal)	1379	445	1553	597	1319	363	1250	222	0.05
Energy intake from snacks and beverages (kcal)	173	122	243	157	157	88	111	52	0.001

**Table 4:** Association between Japanese Food Guide Spinning Top score at baseline (T1) and change in BAZ and HAZ (T2-T1).

Variable	JFGST Score at Baseline (T1)					
	Low		Middle		High	
	Coef.	95%CI	Coef.	95% CI	Coef.	95% CI
β(95% CI) for change in BAZ (T2-T1)	Ref.		0.16	(−0.29–0.60)	−0.14	(−0.61–0.33)
β(95% CI) for change in HAZ (T2-T1)	Ref.		−0.15	(−0.50–0.21)	−0.06	(−0.43–0.30)

BAZ, body mass index for age z-score; HAZ, height for age z-score; CI, confidence interval; JFGST score, Japanese Food Guide Spinning Top score; Ref., reference.

**Table 5:** Distribution of the intakes of each dish category according to dietary intake level of each dish at baseline (T1) and association between dietary intake of each dish (T1) and change in BAZ and HAZ (T2-T1).

Variable	Dietary Intake of Each Dish at Baseline (T1)					
	Low		Middle		High	
	Mean/Coef.	SD/95%CI	Mean/Coef.	SD/95%CI	Mean/Coef.	SD/95%CI
Initial intake (mean (SD)) at T1						
Grain dishes (serving/d)	2.0	0.5	2.8	0.2	3.7	0.5
Vegetable dishes (serving/d)	1.1	0.3	2.2	0.3	4.0	1.6
Fish and meat dishes (serving/d)	2.2	0.5	3.3	0.3	4.7	0.8
Milk (serving/d)	0.7	0.4	1.9	0.2	3.8	1.4
Fruits (serving/d)	0.3	0.1	0.6	0.1	1.1	0.3
Total energy intake (kcal/d)	980	120	1285	85	1886	391
Energy intake from snacks and beverages (kcal/d)	61	22	145	24	317	98
β(95% CI) for change in BAZ (T2-T1)						
Grain dishes (serving/d)	Ref.		−0.08	(−0.60–0.44)	−0.16	(−0.82–0.49)
Vegetable dishes (serving/d)	Ref.		0.13	(−0.36–0.61)	−0.29	(−0.82–0.24)
Fish and meat dishes (serving/d)	Ref.		0.32	(−0.17–0.81)	0.49	(−0.06–1.05)
Milk (serving/d)	Ref.		0.39	(−0.08–0.86)	−0.10	(−0.63–0.43)
Fruits (serving/d)	Ref.		−0.03	(−0.50–0.43)	−0.41	(−0.89–0.07)
Total energy intake (kcal/d)	Ref.		0.06	(−0.41–0.52)	0.56	(0.02–1.10)
Energy intake from snacks and beverages (kcal/d)	Ref.		−0.30	(−0.82–0.23)	−0.62	(−1.29–0.06)
β(95% CI) for change in HAZ (T2-T1)						
Grain dishes (serving/d)	Ref.		−0.12	(−0.53–0.30)	0.00	(−0.53–0.52)
Vegetable dishes (serving/d)	Ref.		0.14	(−0.25–0.53)	0.05	(−0.37–0.47)
Fish and meat dishes (serving/d)	Ref.		−0.39	(−0.78–0.01)	−0.53	(−0.97–−0.09)
Milk (serving/d)	Ref.		−0.12	(−0.50–0.26)	0.04	(−0.39–0.47)
Fruits (serving/d)	Ref.		0.12	(−0.25–0.50)	0.25	(−0.13–0.64)
Total energy intake (kcal/d)	Ref.		−0.05	(−0.42–0.32)	−0.32	(−0.75–0.11)
Energy intake from snacks and beverages (kcal/d)	Ref.		0.11	(−0.31–0.53)	0.36	(−0.18–0.90)

BAZ, body mass index for age z-score; HAZ, height for age z-score; CI, confidence interval; Ref., reference. Adjusted for dietary intake of all dishes.

## Supplementary Materials

The following are available online at https://www.mdpi.com/2072-6643/12/5/1290/s1, Table S1: Classification of the food items indicated in the brief-type self-administered diet history questionnaire for children aged three to six years (BDHQ3y) into food groups and dish categories.
