# Supplementary material for: Change in Growth and Diet Quality Among Preschool Children in Tokyo, Japan
Source: Nutrients. 2020 May 1;12(5):1290. doi: 10.3390/nu12051290 (PMC7282265; doi:10.3390/nu12051290)
Supplement: Supplementary file 1 [file nutrients-12-01290-s001.pdf]

Table S1. Classification of the food items indicated in the brief-type self-administered diet history questionnaire for children aged 3-6 years (BDHQ3y) into food groups and dish categories.

Dish category	Food group	Food item
Grain dishes	Cereals	Rice; Rice with sprinkle; Germinated/unrefined/multigrain/wheat-blended rice; Buckwheat noodles; Japanese wheat noodles; instant noodles and Chinese noodles; spaghetti and macaroni; Breads (including white bread and Japanese bread with a sweet filling)
Vegetable dishes	Potatoes	Potatoes (including potatoes, sweet potatoes, taro, yam, other potatoes); french fries and potato chips
	Vegetables	Carrots and pumpkins; tomatoes (including boiled tomato and stewed tomato); green leafy vegetables including broccoli; salted green and yellow vegetable pickles; raw vegetables used in salad (cabbage and lettuce); cabbage and Chinese cabbage; radishes and turnips; other root vegetables (onions, burdock and lotus root); other salted vegetable pickles; mushrooms (all varieties); seaweeds (all varieties)
	Fruit and vegetable juice	0.225 of 100% fruit and vegetable juice
Fish and meat dishes	Beans	Tofu (i.e. soybean curd) and tofu products; natto (i.e. fermented soybeans); miso for miso soup
	Fish and shell fish	Dried fish and salted fish (including salted mackerel, salted salmon and dried horse mackerel); small fish with bones; canned tuna; oily fish (including sardines, mackerel, saury, amberjack, herring, eel and fatty tuna); non-oily fish (including salmon, trout, white meat fish, freshwater fish and bonito); squid, octopus, shrimp and clam; fish meat paste products
	Meat	Chicken (including ground chicken); pork and beef (including ground pork and beef); liver; ham, sausages and bacon
	Egg	Eggs
Milk	Dairy Products	Full-fat milk; low-fat milk; yoghurt and yoghurt drink; cheese; ice cream
Fruits	Fruits	Citrus fruit including oranges, strawberries; persimmons and kiwi fruit; other fruits; jam
	Fruit and vegetable juice	0.775 of 100% fruit and vegetable juice
Snacks and beverages	Confectionaries	Rice crackers, rice cakes and Japanese-style pancakes; Japanese sweets; cakes, cookies and biscuits; snacks; chocolates
	Soft drinks	Cola and sweetened soft drinks (including sports drinks); cocoa; lactic acid bacteria beverages; fruit juice excluding 100% juice

Note: In BDHQ3y, 100% fruit and vegetable juice was asked as one food item, so when we categorize 100% fruit and vegetable juice into vegetable group and fruit group, it was classified by multiplying by each coefficient.
